# Transductive meta-learning with enhanced feature ensemble for few-shot semantic segmentation

**DOI:** 10.1038/s41598-024-54640-6

**Published:** 2024-02-18

**Authors:** Amin Karimi, Charalambos Poullis

**Affiliations:** Immersive and Creative Technologies Lab, Department of Computer Science and Software Engineering, Concordia University, Montreal, Canada

**Keywords:** Computer science, Computational science

## Abstract

This paper addresses few-shot semantic segmentation and proposes a novel transductive end-to-end method that overcomes three key problems affecting performance. First, we present a novel ensemble of visual features learned from pretrained classification and semantic segmentation networks with the same architecture. Our approach leverages the varying discriminative power of these networks, resulting in rich and diverse visual features that are more informative than a pretrained classification backbone that is not optimized for dense pixel-wise classification tasks used in most state-of-the-art methods. Secondly, the pretrained semantic segmentation network serves as a base class extractor, which effectively mitigates false positives that occur during inference time and are caused by base objects other than the object of interest. Thirdly, a two-step segmentation approach using transductive meta-learning is presented to address the episodes with poor similarity between the support and query images. The proposed transductive meta-learning method addresses the prediction by first learning the relationship between labeled and unlabeled data points with matching support foreground to query features (intra-class similarity) and then applying this knowledge to predict on the unlabeled query image (intra-object similarity), which simultaneously learns propagation and false positive suppression. To evaluate our method, we performed experiments on benchmark datasets, and the results demonstrate significant improvement with minimal trainable parameters of 2.98*M*. Specifically, using Resnet-101, we achieve state-of-the-art performance for both 1-shot and 5-shot Pascal-$5^{i}$, as well as for 1-shot and 5-shot COCO-$20^{i}$.

## Introduction

Deep neural networks can learn rich information about visual features of classes that appear in images when trained on vast amounts of labeled data. These attributes significantly contributed to various critical applications, including medical applications^1,2^.However, their ability to generalize to new classes diminishes when presented with only a limited number of labeled examples^3^, which is a prevalent issue in domains such as geospatial and medical, where collecting and labeling large datasets is a complex and expensive process. To overcome this issue, researchers have proposed the few-shot learning paradigm, which attempts to mimic the capacity of the human visual system to rapidly learn new classes from a small number of labeled examples.

**Figure 1:**
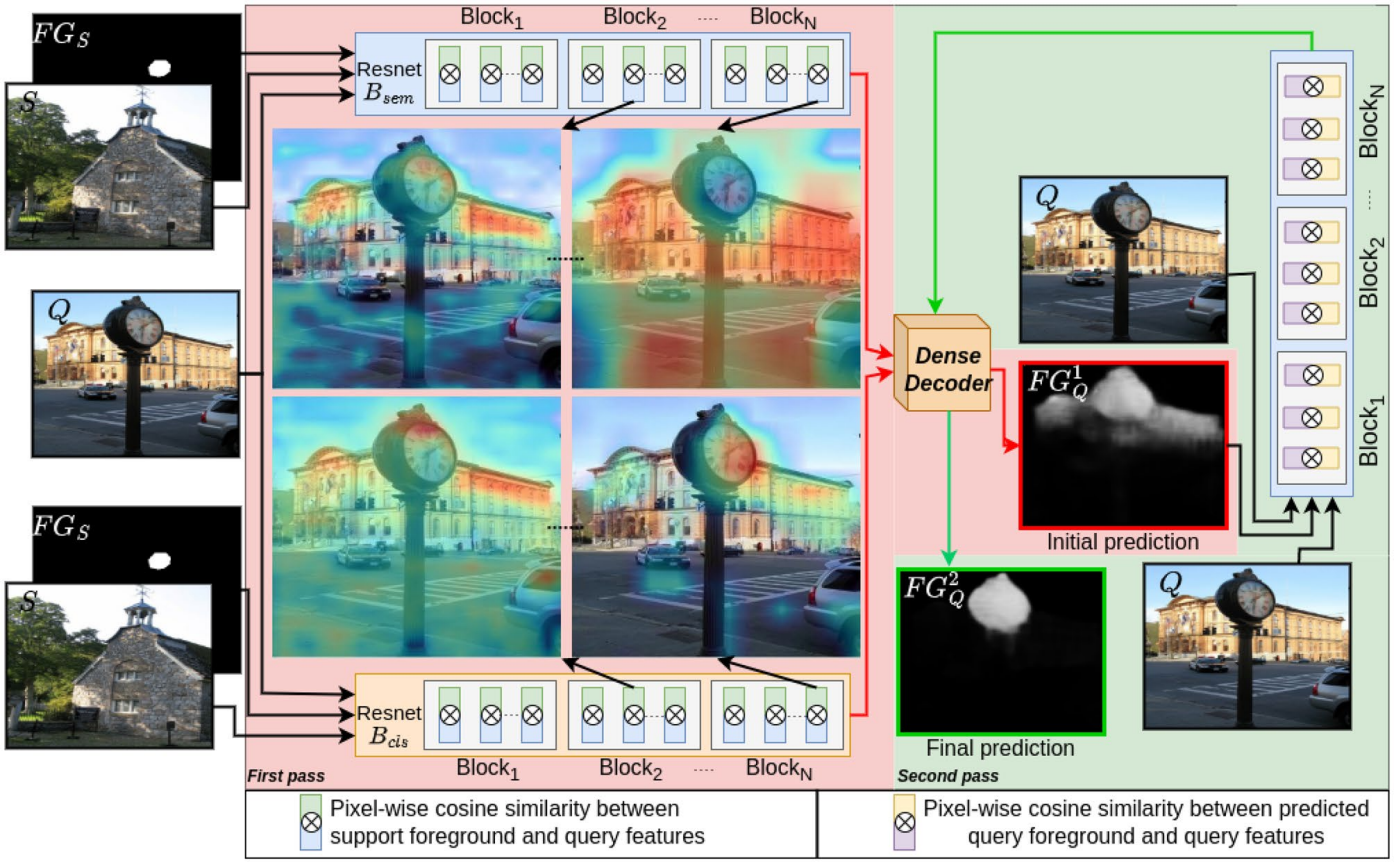
We propose two-pass end-to-end method for few-shot semantic segmentation. The approach leverages an ensemble of visual features learned from pretrained classification $B_{cls}$ and semantic segmentation $B_{sem}$ networks with the same architecture. $B_{sem}$ is also used as a base class extractor. The first pass (red background) matches support foreground features to query features to address intra-class variation, and the second pass (green background) suppresses false positives and propagates query foreground features to leverage intra-object variation. Heatmaps show pixel-correlations between the query features and support foreground features in different layers of the network.

This paper focuses on few-shot semantic segmentation, a special case of semantic segmentation in which the model must generalize to novel(unseen) classes and classify the pixels in an image. The most challenging aspect of few-shot segmentation is fully utilizing the information in the small support set of training examples *K* on *N* unseen classes (*N*-way, *K*-shot for $K<5$). Two primary strategies for fewshot image understanding are proposed. The first strategy centers on the learning-to-learn (or meta-learning) paradigm. In order to simulate the tasks that will be presented during inference, meta-learning strategies popularized the necessity of organizing training data into episodes^4–16^. Similar to standard training, the second line of research addresses few-shot image understanding by training a network using base classes and fine-tuning with novel classes^17–24^. A frozen pretrained classification backbone is utilized by the both line of researches because it has been demonstrated to generalize more effectively to unseen classes (Fig. 1).

**Figure 2:**
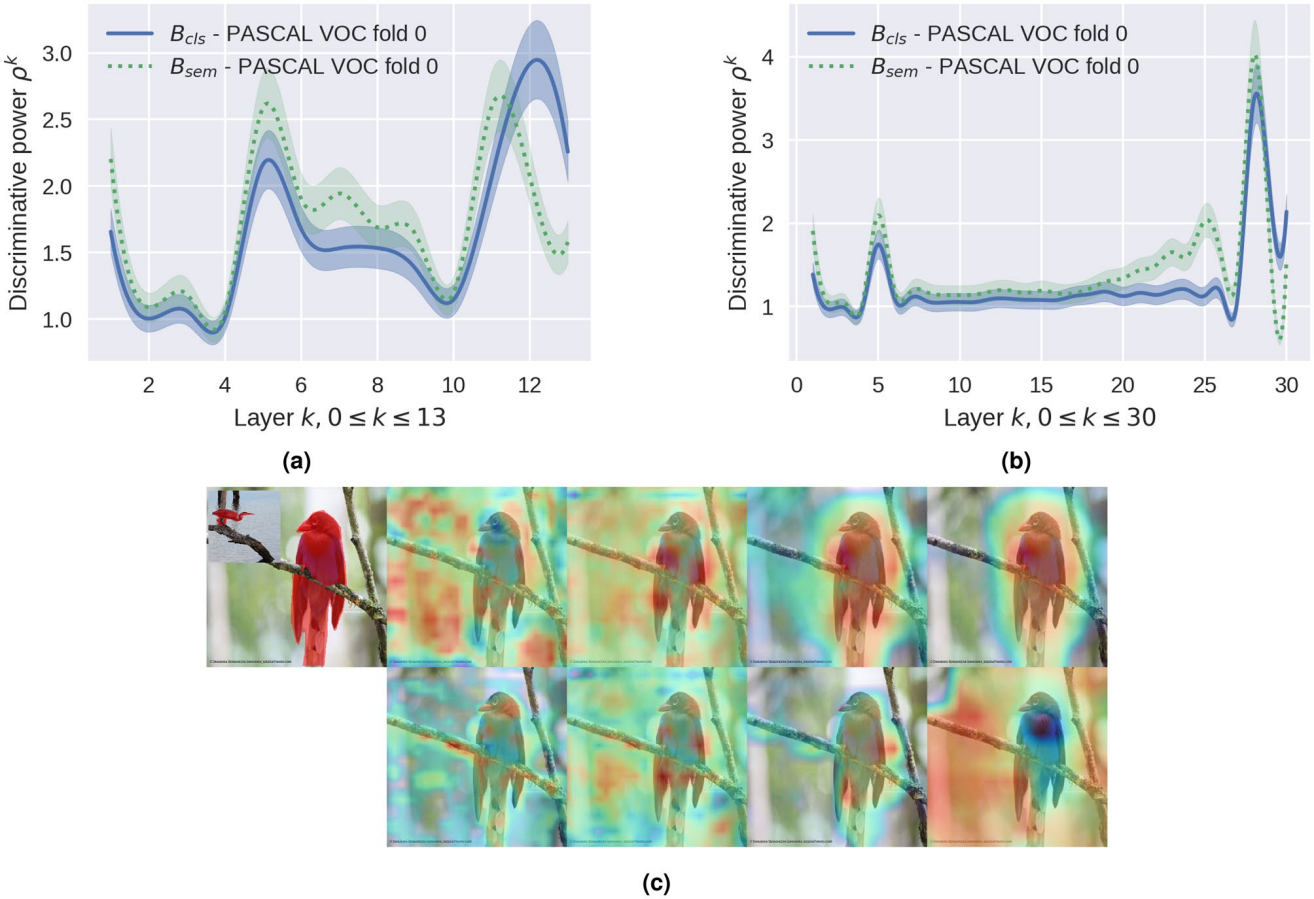
Discriminative power of classification vs semantic segmentation networks. (**a**) : classification network (Resnet-50), : semantic segmentation network (Resnet-50). The discriminative power $\rho ^{k}$ at layer *k* is measured as the ratio $\rho ^{k} = \frac{\frac{1}{N} \sum _{i}^{N} cos\left( FG_{Q}^{i},P_{S}\right) }{\frac{1}{M} \sum _{j}^{M} cos\left( BG_{Q}^{j},P_{S}\right) }$ (**b**) Same as (**a**) but for Resnet-101. Graphs for all folds are in the appendix. (**c**) The top left shows the query image, with an inset of the corresponding support image. The remaining panels depict pixel-correlations between the query features and support foreground features in different layers (from left to right, intermediate layers to final layers) of a pretrained classification (top row) and semantic segmentation networks (bottom row). The discriminative power of a semantic segmentation network is higher at intermediate layers, and the discriminative power of a classification network is higher at the final layers as also demonstrated in (**a**).

The first observation is that a pre-trained classification backbone on a large-scale dataset such as Image-Net contains rich semantic clues; however, it is suboptimal to adopt directly for a segmentation task^9^. Nevertheless, the majority of recent techniques have shown that fine-tuning a pre-trained classification backbone during the episodic training phase is susceptible to overfitting. Experiments presented in^9^ to fine-tune the entire backbone or a subset of layers in FSS demonstrate a negative effect on the final result. Consequently, during the episodic phase, updating millions of backbone parameters necessitates careful training considerations and increases the demand for training resources and time. The recent work to address this issue^9^, which achieves state-of-the-art with a Resnet-50, significantly increases the memory requirements compared to other few-shot techniques. The objective of updating the backbone is to provide enhanced pixel-level features, which is an open problem in FSS. To achieve enhanced pixel-level features without fine-tuning the backbone, we investigated the distinctions between a classification and segmentation backbone. Classification networks are trained with image-level labels and learn visual features that incorporate the spatial distribution and shape of the objects at a higher level of abstraction. In contrast, semantic segmentation networks trained on pixel-level labels discover visual features at the pixel-level that incorporate contextual information based on the spatial relationships between different objects in the image^25–28^. In other words, the discriminative power of a semantic segmentation network is higher at intermediate layers, while a classification network has a higher discriminative power at the final layers. We present the experiments and analysis on the impact that a pretrained backbone can have on the pixel-wise feature correlations, when it is pretrained on a classification versus a semantic segmentation task. For the comparison, we used a frozen classification backbone pretrained on ImageNet-1K and a frozen semantic segmentation backbone pretrained on base classes as described in^10^. We analyzed the pixel-correlations between the query features and support foreground features by calculating the discriminative power of the features at each backbone layer. The discriminative power $\rho ^{k}$ at layer *k* is measured as the ratio $\rho ^{k} = \frac{\frac{1}{N} \sum _{i}^{N} cos\left( FG_{Q}^{i},P_{S}\right) }{\frac{1}{M} \sum _{j}^{M} cos\left( BG_{Q}^{j},P_{S}\right) }$ where $P_{S}$ is the support prototype calculated by averaging all the foreground support features $FG_{S}$. The numerator is the average cosine distance of the *N* foreground query features $FG_{Q}^{i}, 0 \le i \le N$ to the foreground support prototype $FG_{S}$, and the denominator is the average cosine distance of the *M* background query features $BG_{Q}^{j}, 0 \le j \le M$ to $FG_{S}$. Intuitively, the higher the ratio $\rho ^{k}$ the higher the discriminative power to differentiate between the query foreground and background features w.r.t. the support foreground features at layer *k*. Figure 2a and b show the discriminative power calculated using more than 4 000 episodes from Pascal-$5^{i}$, $\rho ^{k}$ of each backbone at layers $k, 1 \le k \le |B_{cls}|$ of the frozen pretrained backbones $B_{cls}$ and $B_{sem}$. Figure 2c top-left, shows the query image, with an inset of the corresponding support image. The remaining panels depict pixel-correlations between the query features and support foreground features in different layers (from left to right, intermediate layers to final layers) of a pretrained classification network (top row) and a semantic segmentation network (bottom row) which shows the higher discriminative power of the semantic segmentation network in the intermediate layers and similarly, for the classification network in the final layers. Utilizing the advantages of each, we present a multi-scale feature ensemble comprised of visual features learned by pretrained classification and semantic segmentation networks to specifically satisfy the need for both rich semantic cues and pixel-level information.

The second observation is that the object in the support image is frequently not visually similar to that in the query image. Several factors contribute to this, including viewpoint variation, illumination changes, scale, deformation, occlusion, intra-class variation, clutter, and motion. Consequently, query segmentation may contain some errors. Numerous techniques for self-refinement based on initial query prediction have been proposed^7,11,16,29^. Recently^7^, proposed a two-step segmentation method by utilizing the high confidence area of initial query prediction via non-differentiable thresholding, which has a number of limitations. In contrast, we present a two-pass end-to-end dense correlation learning method that enables the network to learn the visual disimilarities between the query foreground features and the false positives without introducing non-differentiable operations or additional components. In the first pass, intra-class similarity is addressed by matching support foreground features to query features, and in the second step, intra-object similarity is addressed by suppressing false positives from the initial prediction and propagating query foreground features throughout the query image. The proposed method does not introduce any additional trainable parameters to the network, whereas the^7^ fine-tunes the last two blocks of a backbone. Moreover, our self-refinement module can operate on top of any backbone, which is another significant advantage over^7^ which reshapes embedding space for self-refinement.

The third observation is that false positives account for a substantial proportion of incorrect classifications and significantly hinder performance. As noted by^10^, the presence of base classes in the background of the query image can lead to false positive predictions, as the network may incorrectly classify pixels that are not part of the object of interest. To address this issue, they proposed auxiliary layers on top of a base learner that is trained on base classes to predict whether or not each pixel in the output of the meta learner corresponds to a base class. By using this information to selectively mask out base class predictions, they were able to reduce the number of false positives and improve segmentation accuracy. Inspired by this work and based on observations from our extensive experimentation -as described in the appendix- we propose a method that reduces false positives caused by base classes that is both simpler and faster than the method proposed by^10^, resulting in a shorter training time with the same functionality and performance.

In this paper, we present a two-pass end to end method for few-shot semantic segmentation that addresses each of the aforementioned issues. The proposed method (Fig. 1) leverages an ensemble of visual features learned by segmentation and classification backbones to segment a query image in two steps. Dense convolutional layers trained to match support objects to query images using ensemble features in first step and propagate initial query predictions in the second step.

We evaluate our method on the benchmark datasets Pascal-$5^{i}$ and COCO-$20^{i}$, and report our results. On Pascal-$5^{i}$ 1-shot and 5-shot, with a Resnet-101 backbone, we achieve state-of-the-art by a margin of $2.51\%$ and $1.12\%$, respectively. Similarly, on COCO-$20^{i}$ 1-shot and 5-shot, with a Resnet-101 backbone, we achieve state-of-the-art by a margin of $3.98\%$ and $1.6\%$, respectively. Our model has a minimal number of trainable parameters i.e. 2, 980, 711 compared to the baseline^29^ i.e. 2, 587, 394.

The paper is organized as follows: Section “Related work” outlines the most recent and pertinent work in few-shot semantic segmentation. In Section “Methodology”, the methodology is described in depth, followed by the experiments and ablations on the two benchmark datasets Pascal-$5^{i}$ and COCO-$20^{i}$ in Section “Experiments”. We conclude and provide suggestions for future work in Section “Conclusion”.

## Related work

Few-shot learning techniques enable learners to generalize to new classes using a small number of labeled samples. These techniques follow a similar pipeline: a pre-trained backbone network is used to generate feature embeddings from input images, and a model head is used to generate segmentation maps using these embeddings as input. Numerous techniques have been proposed that fall into one of four broad categories: (i) metric learning techniques where the objective is to learn a mapping from image space to feature space that ensures the distance between feature vectors of similar categories is small, while it is large for dissimilar categories^11,30–33^, (ii) initialization-based techniques where the objective is to learn a good model initialization so that fine-tuning is possible with a few training examples and a small number of gradient update steps^34–41^, (iii) Hallucination-based techniques where the objective is to learn a generator from the available data that “hallucinates” novel class data for data augmentation^42,43^, (iv) semantic-based learning techniques where the objective is to learn a generator conditioned on additional attributes, typically semantic word embeddings. Then, a layer for fine-tuning classification is applied to features from both types of classes^44–46^.

Our work falls into the metric-based techniques and is trained with episodic training as proposed by initialization-based approaches. Early work with metric-based approaches used a two branch network to find the most similar area in the query image using extracted support prototypes based on distance measures, such as Euclidean distance and cosine distance^11^. Other work proposed additional modules to compare query pixels and support prototypes^12^, while others focused on the limited representation capability of a single prototype and proposed methods to develop multiple prototypes to perform comparisons^13–16^.

Recently^17–21^, reevaluated the use of cross-entropy for training the network on base classes and demonstrated that competitive performance could be attained through fine-tuning on unseen classes. Following this pattern, works including^18,22,23^ demonstrated that transductive few-shot learning could enhance performance. Specifically^24^, attained competitive performance by incorporating transductive loss terms into the training and then fine-tuning a single classifier layer trained on base classes. Shannon entropy^18,24^ on each query sample and KL divergence on background/foreground distribution of samples^24^ are the two most common transductive losses. This research demonstrated that transductive learning could not generalize to a new class, however, it can learn the characteristics of a specific sample of a new category, substantially improving the final results.

Currently, few-shot semantic segmentation techniques tend to use all available information and learn the visual similarities between the pixels in the query and support image. Particularly, all-pairs field transforms introduced by^47^ for visual similarities contributed to further considerable gains in few-shot semantic segmentation. The authors of^29^ recast few-shot semantic segmentation as a visual similarity task and perform $N^4$ all-pairs visual comparisons between the pixel-level features in the query and support images. Instead of learning similarities between class prototypes, their network is trained on the visual similarities between all pixel pairings at various network layers.

Several methods have recently shown a considerable performance improvement using pre-trained transformer backbone. Shi et al.^8^ suggested a method for computing similarities between query pixels and all support pixels using a multi-level pixel-wise attention module. The authors reported a substantial improvement when employing a pre-trained transformer backbone as opposed to a convolutional backbone such as ResNet. Zhang et al.^48^ revives the framework of using a backbone for feature extraction followed by a linear classification head. The authors propose a transformer as the backbone and a classification head that combines pixel-level and class-level features, which has been shown to capture global context better than a convolutional network, significantly boosting performance. Recent works such as^8^, have demonstrated significant gains in performance, however, this can easily be attributed to the vision transformer backbone rather than the effectiveness of their proposed technique.

Despite these advancements, there are still challenges to overcome, most notably the bias towards the base classes and insufficient visual similarity between the support and query image, which can result in subpar performance. Our method alleviates these issues. Specifically, our technical contributions are as follows:

- An end-to-end transductive learning method for few-shot semantic segmentation. Uniquely, the matching operates on dense, multi-level visual similarities between support-query pixels and query-query pixels in the first and second passes, respectively.
- A feature ensemble comprised of visual features learned by pretrained classification and semantic segmentation networks. Furthermore, the semantic segmentation network, through the use of a simple and efficient pipeline, serves as a base class and background extractor, drastically reducing false positives, as shown in Table 1

**Table 1:**
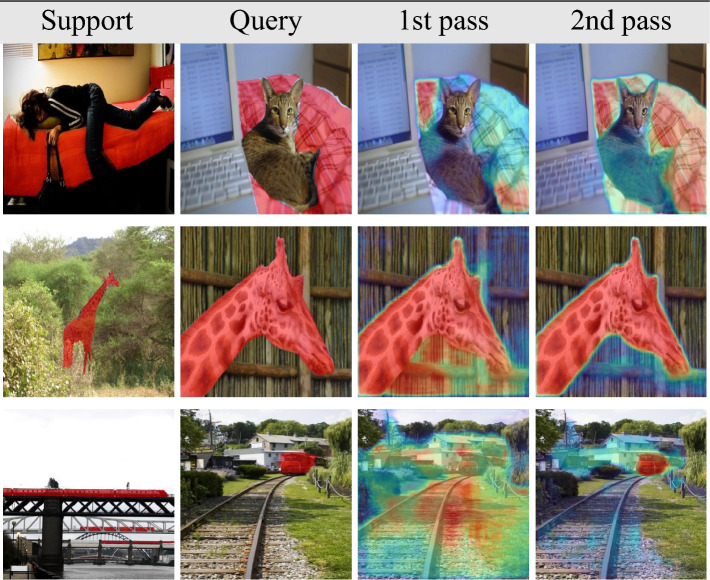
Results from our two-pass method. 1st pass: intra-class similarity ($S\longrightarrow Q$). 2nd pass: intra-object similarity ($Q\longrightarrow Q$).

.
- Our method, using Resnet-101 backbone, achieves state-of-the art performance on 1-shot and 5-shot Pascal-$5^{i}$ as well as COCO-$20^{i}$ while requiring only 2.98*M* in trainable parameters.

## Methodology

The input is a pair of images of the same class *S* and *Q* which form the support and query respectively.

### Learning intra-class similarity $S \rightarrow Q$

The first pass takes the support $S_{1}=S$ and query *Q* as inputs. The objective of this pass is to learn intra-class similarity by learning features from the support $S_{1}$ and segment visually similar features in the query *Q*.

A backbone $B_{cls}$ is a frozen pretrained classification network that learns features $f_{S_{1}}^{cls}$ and $f_{Q}^{cls}$ for image $S_{1}$ and *Q*, respectively. These features encode the spatial distribution and shape of the objects at a more abstract level. This information is supplemented by features $f_{S_{1}}^{sem}$ and $f_{Q}^{sem}$ learned by a backbone $B_{sem}$, a frozen semantic segmentation network trained on both background and base classes. The training of $B_{sem}$ with pixel-level labels results in features $f_{S_{1}}^{sem}$ and $f_{Q}^{sem}$ capturing contextual information and spatial similarities.

Support features from the two backbones, $f_{S_{1}}^{cls}$ and $f_{S_{1}}^{sem}$, are multiplied by the foreground mask $FG_{S_{1}}$ in order to remove background-related features.

1 $$\begin{aligned} f_{S_{1}}^{cls}&= FG_{S_{1}} \otimes B_{cls}(S_{1}), \, f_{Q}^{cls} = B_{cls}(Q) \end{aligned}$$

2 $$\begin{aligned} f_{S_{1}}^{sem}&= FG_{S_{1}} \otimes B_{sem}(S_{1}), \, f_{Q}^{sem} = B_{sem}(Q) \end{aligned}$$

Next, we compare the support and query features by computing the cosine similarity between all pairs of pixels in $f_{S_{1}}^{i}$ and $f_{Q}^{i}$, where $i \in \{cls, sem\}$. This is performed at different depths of each backbone leading to a set of multi-scale 4D volumes, each given by,

3 $$\begin{aligned} HV\left( f_{S}^{i}, \,f_{Q}^{i}\right) = \text {ReLU}\left( \frac{f_{S}^{i} \cdot f_{Q}^{i}}{\left| f_{S}^{i}\right| \cdot \left| f_{Q}^{i}\right| }\right) , \end{aligned}$$

where $i \in \{cls, sem\}$. For features with dimensions in ${\mathbb {R}}^{C\times H\times W}$ the dimensions of the volume are ${\mathbb {R}}^{C\times H\times W \times H \times W}$ , where *C* is the number of channels, and *H*, *W* are the height and width, respectively. This module does not have any trainable parameters.

4D convolutions are applied on the set of multi-scale hypercorrelation volumes. This module, adapted from^29^, applies the 4D convolutions on center-pivot pixels to reduce the memory and time requirements. Incrementally, lower scale features are upsampled and concatenated with higher scale features, followed by average pooling on the last two dimensions in order to reduce the dimensions of the concatenated correlations ${\mathscr {C}}_{cls}$ and ${\mathscr {C}}_{sem}$ to ${\mathbb {R}}^{C\times H\times W}$.

4 $$\begin{aligned} {\mathscr {C}}_{cls}&= AvgPool\left( Conv^{4D}\left( HV\left( f_{S_{1}}^{cls}, f_{Q}^{cls}\right) \right) \right) \end{aligned}$$

5 $$\begin{aligned} {\mathscr {C}}_{sem}&= AvgPool\left( Conv^{4D}\left( HV\left( f_{S_{1}}^{sem}, f_{Q}^{sem}\right) \right) \right) \end{aligned}$$

The first pass concludes with two decoders $Dec_{1}$ and $Dec_{2}$. $Dec_{1}$ operates on ${\mathscr {C}}_{cls}$ and ${\mathscr {C}}_{sem}$ and for each generates a semantic segmentation mask of the foreground $FG_{i}$ and background $BG_{i}$, where $i \in \{cls, sem\}$, supervised with the losses ${\mathscr {L}}_{cls} = \frac{1}{N} \sum _{n=1}^{N} CE\left( BG_{cls} \oplus FG_{cls}, Q^{gt}_{n}\right)$ and ${\mathscr {L}}_{sem} = \frac{1}{N} \sum _{n=1}^{N} CE\left( BG_{sem} \oplus FG_{sem}, Q^{gt}_{n}\right)$, respectively, where $Q^{gt}_{n}$ is the *n*-th ground-truth query foreground mask, $n \in N$. $Dec_{2}$ transforms the concatenated correlations into $FG_{Q}^{1}$ and $BG_{Q}^{1}$, supervised by loss ${\mathscr {L}}_{combined}$.

6 $$\begin{aligned}&FG_{cls}, BG_{cls} = Dec_{1}\left( {\mathscr {C}}_{cls}\right) \end{aligned}$$

7 $$\begin{aligned}&FG_{sem}, BG_{sem} = Dec_{1}\left( {\mathscr {C}}_{sem}\right) \end{aligned}$$

8 $$\begin{aligned}&FG_{Q}^{1}, BG_{Q}^{1} = Dec_{2}\left( {\mathscr {C}}_{cls} \oplus {\mathscr {C}}_{sem}\right) \end{aligned}$$

where the superscript $(.)^{1}$ indicates an outcome of the first pass. The loss is given by ${\mathscr {L}}_{combined} = \frac{1}{N} \sum _{n=1}^{N} \left[ CE\left( BG_{Q}^{1} \oplus FG_{Q}^{1}, Q^{gt}_{n}\right) - \kappa {\mathscr {L}}_{Sh}\right]$, where $\kappa =0.1$. The second term of $L_{combined}$ is the transductive loss term given by Shannon entropy ${\mathscr {L}}_{Sh}$,

9 $$\begin{aligned} {\mathscr {L}}_{Sh} = \frac{1}{H\times W} \sum _{p=1}^{H\times W} \left( BG_{Q}^{1}(p) \oplus FG_{Q}^{1}(p)\right) log\left( BG_{Q}^{1}(p) \oplus FG_{Q}^{1}(p)\right) \end{aligned}$$

where $p \in H\times W$ is pixel. The Shannon entropy encourages the network to have a polarised initial prediction with a high or low confidence area^49^, which reduces the number of false positives. The impact of transductive terms is explained further in the appendix.

### Learning intra-object similarity $Q \rightarrow Q$

As input for the second pass, the query image *Q* serves as both the support $S_{2}=Q$ and query *Q*. The objective of the second pass is to learn intra-object similarity by propagating in the query image *Q* those features in *Q* that were visually similar to the features of the support $S_{1}$ in the first pass. As mentioned previously, the premise is twofold: (i) that intra-object similarity, which is the visual similarity between features in the same image, is greater than intra-class similarity, which is the visual similarity between features in two different images of the same class, and (ii) that learning features of the background and base classes reduces false positives. It has been demonstrated that the affinity between unlabeled samples has a significant effect on transductive learning^22^. We observed that a pretrained semantic segmentation backbone has greater pixel affinity than a pretrained classification network. In the second pass, we therefore employ a semantic segmentation backbone.

**Figure 3:**
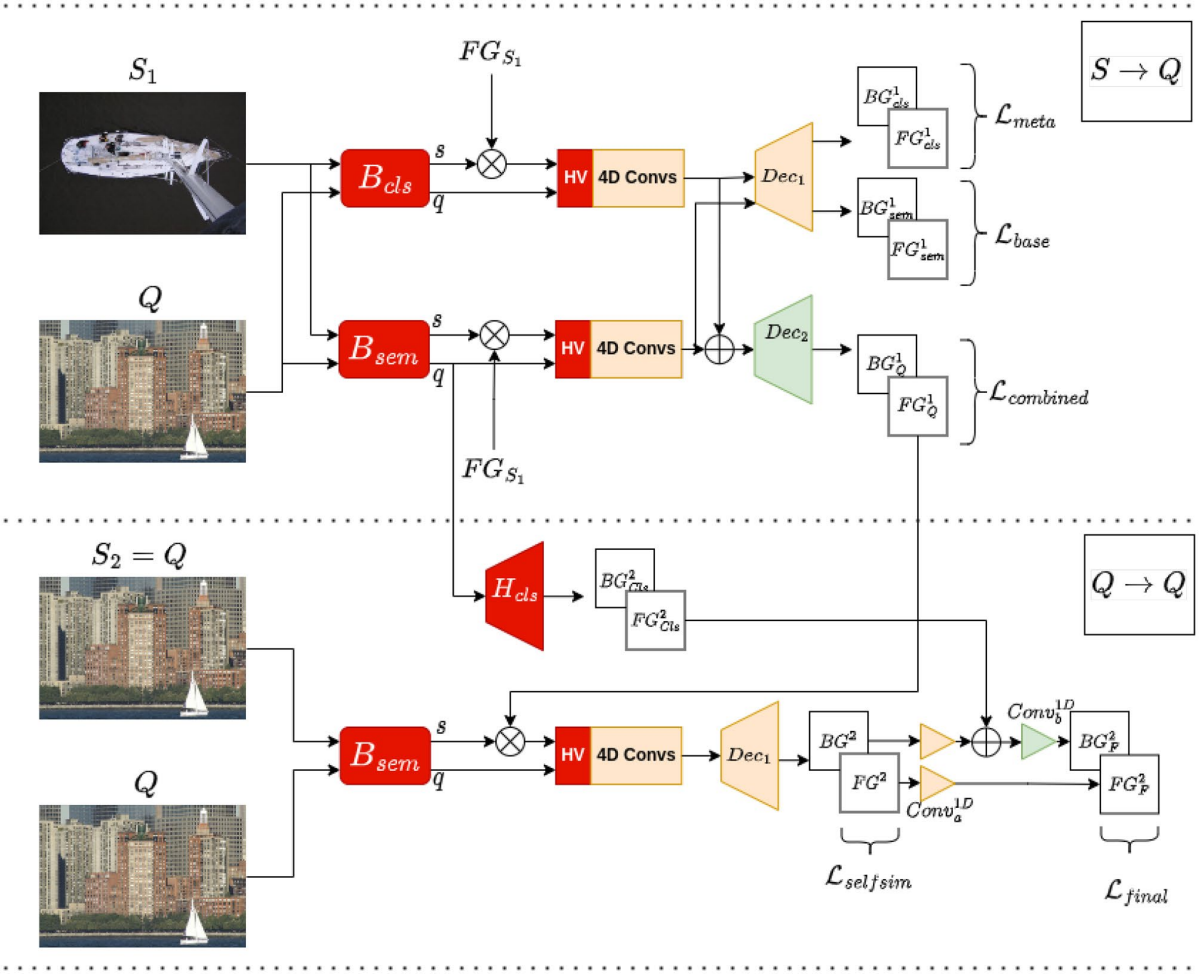
Technical overview of proposed meta-learner. $B_{cls}$, $B_{sem}$: pretrained classification and semantic segmentation networks, respectively (frozen), $H_{Cls}$: pretrained classification layer (frozen), **HV**: Hypercorrelation volumes (multi-scale cosine similarity between features with no trainable parameters), **4D Convs**: 4D convolutions resulting in correlation tensors in ${\mathbb {R}}^{C\times H\times W \times H \times W}$ for feature tensors with dimensions $C\times H\times W$, followed by concatenation across scale and an average pooling on the last two dimensions to reduce the dimensions to ${\mathbb {R}}^{C\times H\times W}$, , : 1D Convolution; the first two $Conv^{1D}_{a}$ share weights, **BG/FG**: Background/Foreground, **Dec**: a decoder; Decoders shown in yellow are the same. Red indicates a frozen module, Orange indicates shared trainable parameters, and Green indicates a module with individually trainable parameters. Total number of trainable parameters: 2.98*M*.

Features $f_{S_{2}}^{2}$ and $f_{Q}^{2}$ are extracted by the semantic segmentation backbone $B_{sem}$. Support features $f_{S_{2}}^{2}$ are multiplied by the foreground mask of *Q* resulting from the first pass. Similar to the first pass, multi-scale hypercorrelation volumes are calculated followed by multi-scale 4D convolutions and average pooling on the last two dimensions. A decoder $Dec_{1}$ maps the correlations ${\mathscr {C}}^{2}$ into $FG^{2}$ and $BG^{2}$ segmentation maps which are supervised with the loss ${\mathscr {L}}_{selfsim} = \frac{1}{N} \sum _{n=1}^{N} CE\left( FG^{2} \oplus BG^{2}, Q^{gt}_{n}\right)$. Each segmentation is then passed through 1D-convolutions sharing weights (shown as  in Fig. 3).

10 $$\begin{aligned}&f_{S_{2}}^{2} = FG^{1}_{Q} \otimes B_{sem}(S_{2}), f_{Q}^{2} = B_{sem}(Q) \end{aligned}$$

11 $$\begin{aligned}&{\mathscr {C}}^{2} = AvgPool\left( Conv^{4D}\left( HV\left( f_{S_{2}}^{2}, f_{Q}^{2}\right) \right) \right) \end{aligned}$$

12 $$\begin{aligned}&FG^2, BG^2 = Dec_{1}\left( {\mathscr {C}}^{2}\right) \end{aligned}$$

where the superscript $(.)^{2}$ indicates an outcome of the second pass.

The semantic segmentation backbone $B_{sem}$, which has been pretrained on background and base classes, serves to eliminate false positives from the query foreground segmentation mask. A pretrained classification layer $H_{cls}$ acting on the backbone’s $B_{sem}$ query features $\left( f_{Q}^{sem}\right) ^{1}$ from the first pass, generates foreground $FG^{1}_{Cls}$ and background $BG^{1}_{Cls}$ maps. The foreground map $FG^{1}_{Cls}$ of the classifier contains base classes. In the penultimate step, the background map of $B_{sem}$, $BG^{2}$, is combined with the foreground map of the classifier $FB^{1}_{Cls}$, passed through a 1D convolution and finally combined with the foreground probabilities of $B_{sem}$, $FG^{2}$. The final map is supervised with loss ${\mathscr {L}}_{final} = \frac{1}{N} \sum _{n=1}^{N} CE\left( FG_{F}^{2} \oplus BG_{F}^{2}, Q^{gt}_{n}\right)$.

13 $$\begin{aligned}&FG^{1}_{Cls}, BG^{1}_{Cls} = H_{cls}\left( \left( f_{Q}^{sem}\right) ^{1}\right) \end{aligned}$$

14 $$\begin{aligned}&FG_{F}^{2} = Conv^{1D}_{a}\left( Conv^{1D}_{b}\left( BG^{2}\right) \oplus FG^{1}_{Cls}\right) \end{aligned}$$

15 $$\begin{aligned}&BG_{F}^{2} = Conv^{1D}_{b}\left( FG^{2}\right) \end{aligned}$$

The proposed meta-learner (Fig. 3) is trained using episodic training supervised by the loss *L* given by,

16 $$\begin{aligned} L = L_{cls} + L_{sem} + L_{combined} + L_{selfsim} + L_{final} \end{aligned}$$

with equal weights for each term.

#### Extension to *K*-shot setting

For *K*-shot setting, we employ the method in^29^. Given *K* support image-mask pairs and a query image, we perform K forward passes to predict *K* masks. Voting is conducted at each pixel location by summing the *K* predictions and dividing each output score by the maximum votes. A pixel is designated as foreground if its voting score exceeds a predetermined threshold.

## Experiments

### Implementation details

#### Modules

 The backbones $B_{cls}$ and $B_{sem}$ are frozen Resnet-style backbones pretrained using supervized classification leanirng on ImageNet-1K and supervized segmentation learning on base classes of each fold respectively. The 4D convolutions all share the same architecture and weights, and have 2.5*M* trainable parameters. There are two decoders having the same architecture. We use episodic training to train the meta-learner with the two frozen backbones $B_{cls}$ and $B_{sem}$.

#### Training

 The training consists of two phases: pretraining and meta-training. Following^10^, we trained a supervised segmentation model on base classes associated with each fold in the first phase. PSPNet with two different backbones, namely ResNet50 and ResNet101, is used as a segmentation model, and it is trained on Pascal-$5^{i}$ for 100 epochs and COCO-$20^{i}$ for 20 epochs, with batch size set to 12 and a stochastic gradient descent optimizer with an initial learning rate $2.5e-3$. In the second phase, the entire model with frozen backbones is trained with episodic learning. In the majority of previous FSS methods, it has been demonstrated that frozen backbone facilitate generalisation in episodic learning. For the Pascal-$5^{i}$ and COCO-$20^{i}$, the batch size is set to 24 and 48, respectively, and the model is trained for 200 iterations using the Adam optimizer and an initial learning rate of $1e-3$. No data augmentation is used during training to ensure a fair comparison with other methods. Four NVIDIA V100 GPUs are employed for training.

### Evaluation

#### Benchmark datasets

We evaluate the performance of the proposed method on two major few-shot segmentation datasets, Pascal-$5^{i}$ and COCO-$20^{i}$, which were constructed from PASCAL VOC 2012 with 20 classes and MS-COCO datasets with 80 classes, respectively. COCO-$20^{i}$ is more challenging because it has more classes, samples, and more object instances per image. With minor modifications to the class partitioning, these two well-known benchmark datasets for semantic segmentation can be utilized to perform few-shot semantic segmentation. Both datasets are partitioned into four folds, with three-quarters of the classes serving as training data (base/seen classes) and the remaining classes serving as validation data (novel/unseen classes). For validation purposes, 1000 episodes of support and query images are sampled from the validation set during the inference phase.

#### Measures

 Results are reported using mean intersection-over-union (mIoU) on individual folds, as well as the average of mIoUs across all folds for both datasets.

#### Results

 Table 2 shows the quantitative evaluation on the four folds of the Pascal-$5^{i}$ dataset. All measures are reported according to their original publications. The highest values are displayed in bold and the second-highest appear underlined. We use Min et al.^29^ as a baseline since it has similar architecture to ours and similar number of trainable parameters. Following the few-shot semantic segmentation literature, we focus our comparisons on methods reporting on the two backbones Resnet-50 and Resnet-101. For the purposes of a fair comparison, values that differ less than $0.35\%$ are considered equivalent. Except for Shi et al.^8^, which is included in the comparisons, recent transformer-based methods on few-shot semantic segmentation cannot be integrated with convolutional backbones and are thus excluded because the performance boost is attributed to the change in architecture rather than the methodology^48,50^. A clear example of this is^8^ which without the Swin-B tranformer backbone the authors report a drop by $5\%$ in the mIoU. Our argument is also supported by the experiments reported by the authors in^8^ where they demonstrate that our baseline^29^ when used with a Swin-B backbone gains an average boost of about $6\%$ on mIoU for COCO-$20^{i}$ 1-shot and 5-shot tasks.

**Table 2:** Comparison with current state-of-the-art for Pascal-$5^{i}$ 1-shot and 5-shot tasks.

Backbone	Method	1-shot					5-shot				
		f0	f1	f2	f3	mIoU	f0	f1	f2	f3	mIoU
Resnet-50	REPRI^24^	60.2	67.0	61.7	47.5	59.1	64.5	70.8	71.7	60.3	66.8
	PFENet^4^	61.7	69.5	55.4	56.3	60.8	63.1	70.7	55.8	57.9	61.9
	ProtRel^5^	65.2	72.9	63.3	61.3	65.7	70.2	75.6	68.9	66.2	70.2
	VAT^6^	67.6	72.0	62.3	60.1	65.5	72.4	73.6	68.6	65.7	70.1
	SSP^7^	60.5	67.8	66.4	51.0	61.4	67.5	72.3	75.2	62.1	69.3
	DCAMA^8^	67.5	72.3	59.6	59.0	64.6	70.5	73.9	63.7	65.8	68.5
	BAM + SVF^9^	**69**.**38**	**74**.**51**	**68**.**80**	63.09	**68**.**95**	**72**.**05**	**76**.**17**	**71**.**97**	**68**.**91**	**72**.**28**
	BAM^10^	68.97	73.59	67.55	61.13	67.81	70.59	75.05	70.79	67.20	70.91
	Baseline-HSNet	64.3	70.7	60.3	60.5	64.0	70.3	73.2	67.4	67.1	69.5
	Ours	68.03	73.69	64.25	**64**.**72**	***67.67***	71.26	75.13	67.75	68.11	***70.56***
Resnet-101	REPRI^24^	59.6	68.6	62.2	47.2	59.4	66.2	71.4	67.0	57.7	65.6
	PPNet^14^	52.7	62.8	57.4	47.7	55.2	60.3	70.0	69.4	60.7	65.1
	PFENet^4^	60.5	69.4	54.4	55.9	60.1	62.8	70.4	54.9	57.6	61.4
	ProtRel^5^	67.8	74.6	65.7	62.2	67.5	70.0	75.9	**71**.**8**	65.8	70.9
	VAT^6^	70.0	72.5	64.8	64.2	67.9	**75**.**0**	75.2	68.4	69.5	72.0
	SSP^7^	60.5	67.8	66.4	51.0	61.4	67.5	72.3	75.2	62.1	69.3
	DCAMA^8^	65.4	71.4	63.2	58.3	64.6	70.7	73.7	66.8	61.9	68.3
	Baseline-HSNet	67.3	72.3	62.0	63.1	66.2	71.8	74.4	67.0	68.3	70.4
	Ours	**71**.**25**	**76**.**19**	**67**.**73**	**66**.**47**	***70.41***	73.85	**77**.**53**	70.72	**70**.**41**	***73.12***

The highest values are in bold, and the second-highest are underlined. Average mIoU is bold italic. See appendix for full-sized table.

With a Resnet-101 backbone, our method is state-of-the-art for both 1-shot and 5-shot. It exceeds the baseline^29^ by $4.21\%$ on 1-shot and $2.72\%$ on 5-shot task. Additionally, its margins for the 1-shot and 5-shot are $2.51\%$ and $1.12\%$, compared to the second-best performing methods. With a Resnet-50 backbone, we achieve results comparable to other methods with similar number of trainable parameters. The most recent work of Sun et al.^9^, which achieves state-of-the-art with a Resnet-50, significantly increases the memory requirements because, according to the authors, it requires 128*G* for a batch 8 (16*G* for one image), which is significantly higher than any other few-shot semantic segmentation technique.

The quantitative evaluation of the four folds of the COCO-$20^{i}$ data set is displayed in the Table 3. We achieve state-of-the-art for COCO-$20^{i}$ with Resnet-101 backbone for both 1-shot and 5-shot. It exceeds the baseline^29^ by $9.68\%$ on 1-shot and by $5.40\%$ on 5-shot. In addition, it has a margin of $3.98\%$ and $1.6\%$ over the second-best performing strategy. Using a Resnet-50 backbone, we achieve second-best performance by a margin of $0.35\%$ on 1-shot and $1.12\%$ compared to the significantly more memory-intensive method of Sun et al.

**Table 3:** Comparison with current state-of-the-art for COCO-$20^{i}$ 1-shot and 5-shot tasks.

Backbone	Method	1-shot					5-shot				
		f0	f1	f2	f3	mIoU	f0	f1	f2	f3	mIoU
Resnet-50	REPRI^24^	32.0	38.7	32.7	33.1	34.1	39.3	45.4	39.7	41.8	41.6
	PFENet^4^	36.5	38.6	34.5	33.8	35.8	36.5	43.3	37.8	38.4	39.0
	ProtRel^5^	42.2	48.9	45.5	44.6	45.3	48.0	55.7	50.7	50.1	51.1
	VAT^6^	39.0	43.8	42.6	39.7	41.3	44.1	51.1	50.2	46.1	47.9
	SSP^7^	35.5	39.6	37.9	36.7	37.4	40.6	47.0	45.1	43.9	44.1
	DCAMA^8^	41.9	45.1	44.4	41.7	43.3	45.9	50.5	50.7	46.0	48.3
	BAM + SVF^9^	** 46.87**	**53**.**80**	48.43	44.78	**48**.**47**	**52**.**25**	57.83	51.97	**53**.**41**	**53**.**87**
	BAM^10^	43.41	50.59	47.49	43.42	46.23	49.26	54.20	51.63	49.55	51.16
	Baseline-HSNet	36.3	43.1	38.7	39.2	39.2	43.3	51.3	48.2	45.0	46.9
	Ours	42.15	53.22	**49**.**05**	**48**.**08**	***48.12***	47.50	**59**.**14**	**53**.**19**	51.16	***52.75***
Resnet-101	PPNet^14^	17.0	18.0	21.0	28.9	21.2	19.1	21.5	23.9	30.1	23.7
	PFENet^4^	34.3	33.0	32.3	30.1	32.4	38.5	38.6	38.2	34.3	27.4
	ProtRel^5^	42.9	50.6	46.8	47.4	46.9	50.7	58.3	**52**.**8**	51.3	53.3
	SSP^7^	39.1	45.1	42.7	41.2	42.0	47.4	54.5	50.4	49.6	50.2
	DCAMA^8^	41.5	46.2	45.2	41.3	43.5	48.0	58.0	54.3	47.1	51.9
	Baseline-HSNet	37.2	44.1	42.4	41.3	41.2	45.9	53.0	51.8	47.1	49.5
	Ours	**45**.**48**	**56**.**47**	**51**.**74**	**49**.**84**	***50.88***	48.87	**61**.**10**	55.58	**54**.**03**	***54.90***

The highest values are in bold, and the second-highest are underlined. Average mIoU is bold italic. See appendix for full-sized table.

Table 4 displays qualitative comparisons using a Resnet-50 backbone. The first and second columns represent the support and query images, while the remaining columns represent the results of SSP^7^, HSNET^29^, DCAMA^8^, BAM^10^, and ours (last column). As can be seen, our method can successfully handle challenging cases in which the object in the support differs visually from the object in the query and the visual similarity between the foreground and background is high, as in the second and fourth rows.

**Table 4:**
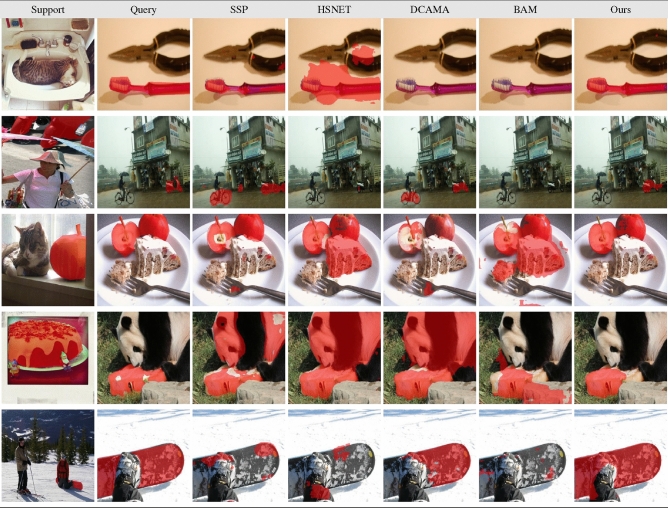
Qualitative results. The first and second columns show the support and query images, respectively, overlaid with the ground truth in red. The remaining columns show the predictions overlaid with a red.

### Ablations

Our method results in a substantial performance increase. We demonstrate this by applying it to the classification-based method of Min et al.^29^. In the subsequent experiments, we use this as a baseline and conduct 32 experiments consisting of a baseline with a classification backbone (with $B_{cls}$), a baseline with a segmentation backbone (with $B_{sem}$), a baseline with dual backbones (with $B_{cls}$ + $B_{sem}$), and a two-pass dual backbone baseline (two-pass with $B_{cls}$ + $B_{sem}$). For each ablation, we use Resnet-50 and Resnet-101 backbones, and conduct experiments on all folds of Pascal-$5^{i}$ for 1-shot and 5-shot. The models are trained for 200 epochs with batch of 12 and Adam optimizer with an initial learning rate of $1e-3$.

We begin with an experiment in which the classification backbone used by the baseline^29^ is replaced with a semantic segmentation network in order to gain a better understanding of the impact that the type of the backbone can have on the performance. The first (Baseline) and second (with $B_{sem}$) rows of each table cell display the results for the 1-shot and 5-shot Pascal-$5^{i}$ tasks, respectively. Using a classification backbone for Resnet-50 is preferable to using a semantic segmentation backbone. The opposite is true for Resnet-101, and this is supported by the outcomes of both 1-shot and 5-shot tasks. As shown in the third row (with $B_{cls}$ + $B_{sem}$), it is evident that using both types of backbone improves performance, which is supported by the results on both tasks. As explained in the introduction, this is due to the fact that the $B_{cls}$ and $B_{sem}$ backbones capture diverse but distinct visual features. The fourth row (two-pass with $B_{cls}$ +$B_{sem}$) displays the results of applying our method to the baseline which increases performance by $3.64\%$ and $3.88\%$ for the 1-shot task with Resnet-50 and Resnet-101 backbones, respectively, and a performance increase of $1.6\%$ and $2.16\%$ for 5-shot for Resnet-50 and Resnet-101, respectively as shown in Table 5. For additional experiments we refer the reader to the “Supplementary Information”.

**Table 5:** Ablations on all components of our method.

Backbone	Method	1-shot					5-shot				
		f0	f1	f2	f3	mIoU	f0	f1	f2	f3	mIoU
Resnet-50	Baseline - HSNET	62.80	70.09	60.16	58.98	63.01	69.12	73.67	66.21	65.44	68.61
	with $B_{sem}$	60.84	68.27	59.70	59.15	61.99	66.85	72.91	66.55	66.02	68.09
	with $B_{cls}$ + $B_{sem}$	64.87	71.79	64.12	60.97	65.44	70.11	74.48	67.51	67.09	69.79
	two-pass with $B_{cls}$ + $B_{sem}$	**66**.**26**	**73**.**76**	**63**.**22**	**63**.**37**	***66.65***	**70**.**55**	**75**.**22**	**67**.**05**	**68**.**02**	***70.21***
Resnet-101	Baseline - HSNET	66.41	71.51	62.30	61.96	65.55	71.45	75.07	67.10	67.60	70.30
	with $B_{sem}$	65.70	72.72	64.01	62.21	66.16	71.16	75.94	69.41	68.03	71.14
	with $B_{cls}$ + $B_{sem}$	68.47	74.02	64.74	63.59	67.71	72.19	76.85	69.76	69.21	72.00
	two-pass with $B_{cls}$ + $B_{sem}$	**69**.**22**	**74**.**49**	**67**.**20**	**66**.**81**	***69.43***	**73**.**28**	**77**.**01**	**69**.**94**	**69**.**64**	***72.46***

Experiments reported for Pascal-$5^{i}$ with Resnet-50 and Resnet-101 backbones. The highest values are displayed in bold and average mIoU is bold italic.

## Conclusion

In conclusion, we proposed a novel two-pass end-to-end method for few-shot semantic segmentation that addresses three key problems affecting performance. The approach leverages an ensemble of visual features learned from pretrained classification and semantic segmentation networks with the same architecture to capture rich and diverse information at different depths. Additionally, the pretrained semantic segmentation network serves as a base class extractor to reduce false positives. The first pass addresses intra-class similarity by matching support foreground features to query features, and the second pass leverages intra-object similarity by learning to suppress false positives and propagating query foreground features. Experimental results on benchmark datasets demonstrate significant improvement in performance with minimal trainable parameters. Specifically, using Resnet-101, the proposed method achieves state-of-the-art performance for both 1-shot and 5-shot Pascal-$5^{i}$, as well as on 1-shot and 5-shot COCO-$20^{i}$.

### Supplementary Information

Supplementary Information.

## Data Availability

The datasets generated and/or analysed during the current study are available in the PASCAL VOC http://host.robots.ox.ac.uk/pascal/VOC/ and COCO https://cocodataset.org/ repositories.
