# Supplementary material for: Transductive meta-learning with enhanced feature ensemble for few-shot semantic segmentation
Source: Sci Rep. 2024 Feb 18;14:4028. doi: 10.1038/s41598-024-54640-6 (PMC10874957; doi:10.1038/s41598-024-54640-6)
Supplement: Supplementary file 1 — Supplementary Information. [file 41598_2024_54640_MOESM1_ESM.pdf]

Transductive Meta-Learning with Enhanced Feature Ensemble for Few-shot Semantic Segmentation (Supplementary Material)

Amin Karimi[1] **and Charalambos Poullis**[1,*]

[1]Immersive and Creative Technologies Lab, Department of Computer Science and Software Engineering, Concordia University
[*]charalambos@poullis.org

ABSTRACT

In the supplementary material, we present (i) additional experiments and explanations on the discriminative power of classification and semantic segmentation networks, (ii) additional justification on employing transductive meta-learning for learning intra-class and intra-object similarity, (iii) additional ablations on false positive reduction, (iv) additional ablations demonstrating the performance boost when using the Shannon entropy loss term $\mathscr{L}_{Sh}$, and (v) larger tables and figures with additional results.

Maximizing Discriminative Power

Figures S1 and S2 show the discriminative power calculated using more than 4,000 episodes from Pascal-5^i, ρ^k of each backbone at layers $k, 1 \leq k \leq |B_{cls}|$ of the frozen pretrained backbones B_{cls} and B_{sem}. Figures S1 shows the results with Resnet-50, and Figure S2 shows the results with Resnet-101. The discriminative power ρ^k at layer k is measured as the ratio $\rho^k = \frac{\frac{1}{N}\sum_i^N cos(FG_Q^i, P_S)}{\frac{1}{M}\sum_j^M cos(BG_Q^j, P_S)}$ where P_S is the support prototype calculated by averaging all the foreground support features FG_S. The numerator is the average cosine distance of the N foreground query features $FG_Q^i, 0 \leq i \leq N$ to the foreground support prototype FG_S, and the denominator is the average cosine distance of the M background query features $BG_Q^j, 0 \leq j \leq M$ to FG_S.

Transductive meta-learning

The object in the support image is frequently not visually similar to that in the query image, leading to under and oversegmentation. Table S1, third column (1st pass) depicts the most frequent cases occurring when matching support foreground features to query features, namely undersegmentation (top), and oversegmentation (middle, bottom). Several strategies have been proposed to use this initial query prediction as an additional source of information to improve results in a second step[1–4]. According to the Gestalt principle, the second step can be utilised to refine an undersegmented initial prediction. However, the issue arises when the initial query prediction yields a large number of false positives. To mitigate these cases, SSP method proposed by[3] presented a two-stage method based on the concept of prototyping for refining the initial query segmentation through the selective propagation of query features in the second step. The selective propagation, which is dependent on a user-defined non-adaptive threshold, eliminates gradients and prevents backpropagation throughout the network. In situations where the probability of false positives is greater than the threshold, this not only fails to suppress them but also makes the problem worse by propagating them. To summarize the advantages of our self-refinement approach over

Instead of introducing non-differentiable operations like hard-thresholding, as in Fan et al., we address this issue by allowing the network to learn the visual disimilarities between the query foreground features and the false positives in an end-to-end manner. In the first pass, support foreground features are matched to query features, and in the second pass, false positives are suppressed and query foreground features are propagated throughout the query image. The proposed second pass does not introduce new parameters to the network. We use multi-level all-pairs field transforms[5] that result in a multiscale hypercorrelation volume[6] to leverage the different levels of visual features learned at each layer of the backbone. Table S1, fourth column (2nd pass) demonstrates some instances of our proposed transductive learning method in which the network simultaneously learns to suppress and propagate from initial segmentation. The advantages of our method of self-refinement are summarized below.

1. We adopted 4D-Conv for our self-refinement module, which outperforms the prototyping approach of SSP.

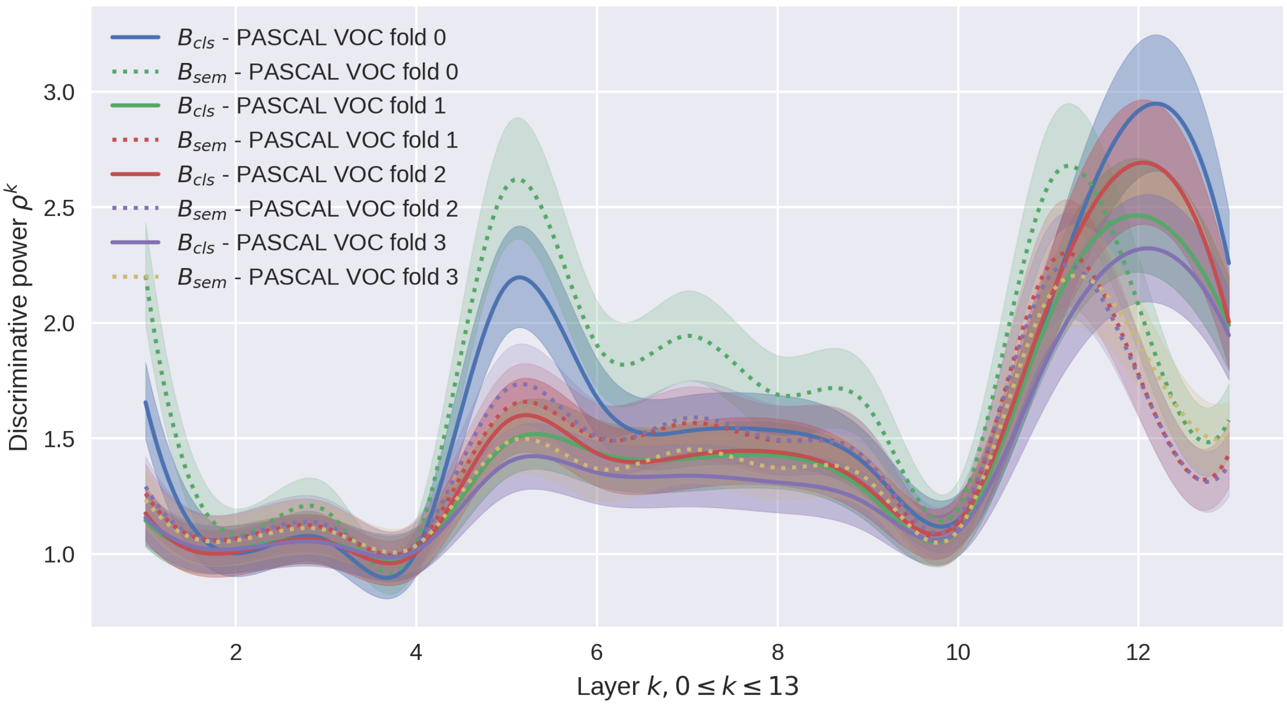

Figure S1. Discriminative power of classification vs semantic segmentation networks. ——: classification network (Resnet-50), - - - -: semantic segmentation network (Resnet-50).

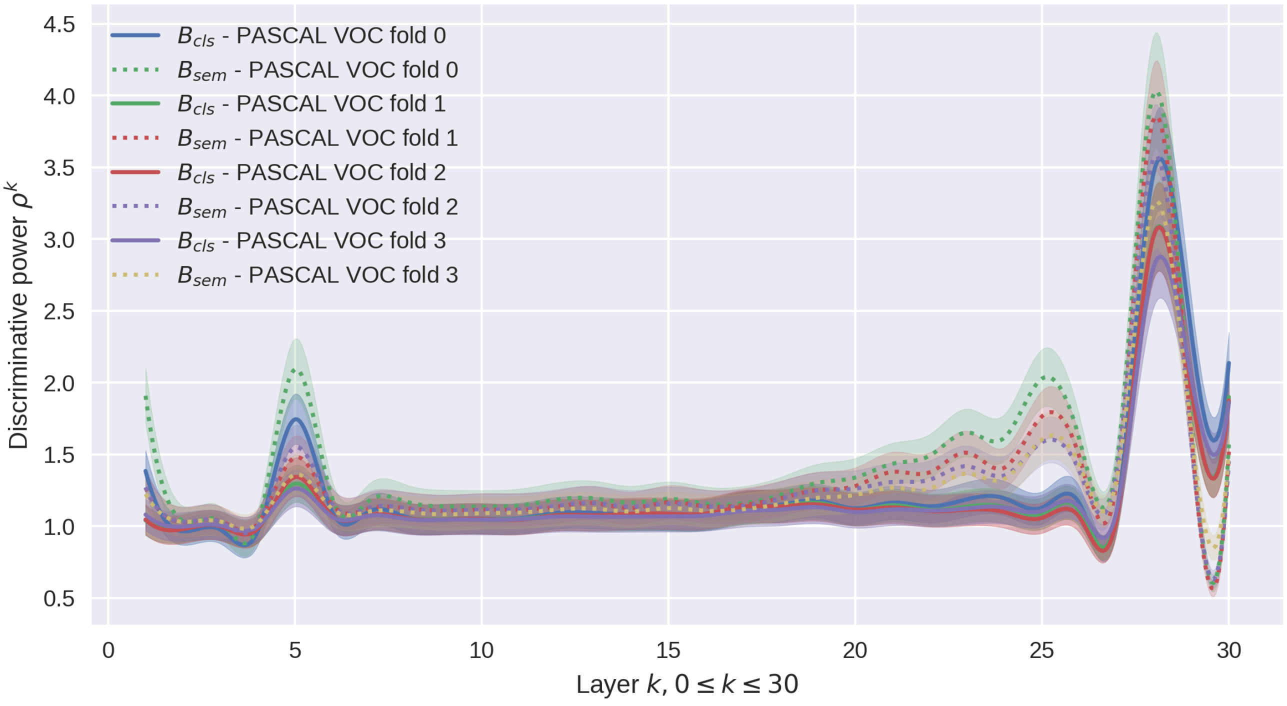

Figure S2. Discriminative power of classification vs semantic segmentation networks. ——: classification network (Resnet-101), - - - -: semantic segmentation network (Resnet-101).

2. Our self-refinement module does not add any additional parameters to the network, whereas the SSP fine-tunes the last two blocks of a ResNet backbone with *1mil parameters*.

3. Our self-refinement module can operate on top of any backbone, which is another significant advantage over SSP which reshapes embedding space for self-refinement.

4. SSP employs a non-differentiable method that uses a user-specified hard-threshold. This restricts the ability to add trainable modules after the non-differentiable operation. We do not use non-differentiable operations. Instead, we enable the network to learn end-to-end the visual differences between the query foreground features and false positives.

Table S1. Results from our two-pass method. 1st pass: intra-class similarity ($S \longrightarrow Q$). 2nd pass: intra-object similarity ($Q \longrightarrow Q$).

Support	Query	1st pass	2nd pass

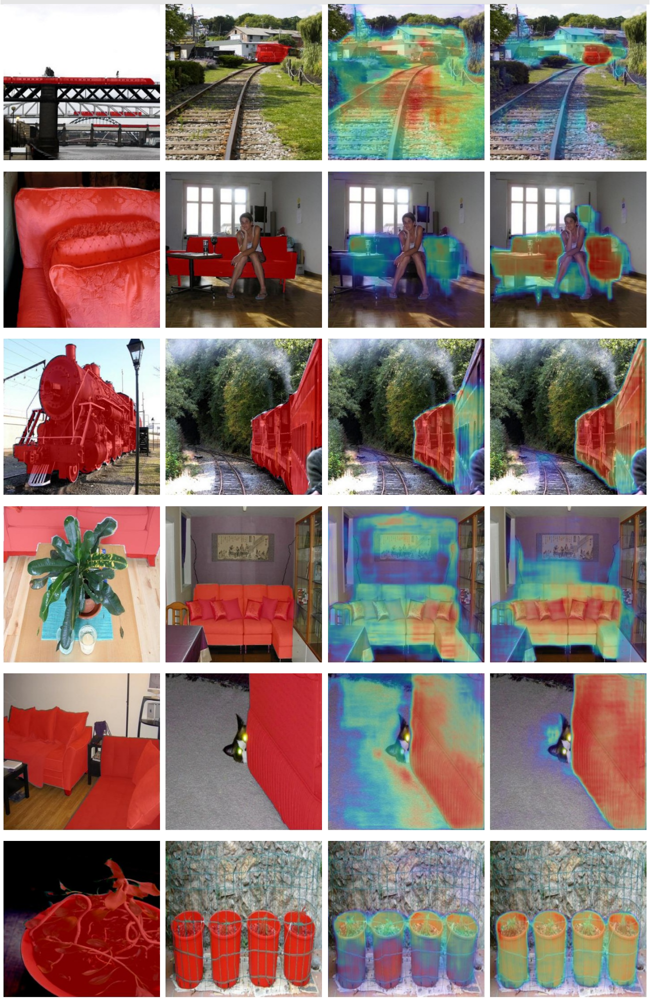

Mitigating Propagation of False Positives

The propagation of false positives can be a significant problem in semantic segmentation, particularly when dealing with complex backgrounds or multiple classes that share similar visual features. As noted by Lang et al.[7], the presence of base classes in the background of the query image can lead to false positive predictions, as the network may incorrectly classify pixels that are not part of the object of interest. To address this issue, they proposed auxiliary layers on top of a base learner that is trained on base classes to predict whether or not each pixel in the output of the meta learner corresponds to a base class. By using this information to selectively mask out base class predictions, they were able to reduce the number of false positives

and improve segmentation accuracy. Specifically, Lang et al. proposed an adjustment factor ψ, namely the Frobenius norm of subtraction of Gram matrices, for integrating the output of the base learner and the meta learner.

$$\psi = ||G_s - G_q||_F \quad (1)$$

$$G = ||f_{low}||.||f_{low}^T|| \in R^{C \times C} \quad (2)$$

$$f_{low}^{S,Q} \in R^{C \times H \times W} \quad (3)$$

where $||.||_F$ is the Frobenius norm, f_{low} is the low-level features, and C, H, W represent the embedding dimension, height, and width of the low-level feature maps, respectively. However, our experiments have shown that the adjustment factor ψ is consistently ignored for combining information. Since the Gram matrix is a product of normalised matrices, its values are in the range $[0,1]$ and as a result, the adjustment factor $\psi \in [0,1]$. Experimentally, we calculated the adjustment factor of thousands of episodes from train and test sets from the entire COCO-20^i and Pascal-5^i datasets, and observed a mean $\mu_\psi < 0.1$ with a variance $\sigma_\psi < .05$. Table S2 shows the results of the experiments on all folds of COCO-20^i and Pascal-5^i datasets. Furthermore, multiplying the network's weights, which are already small, by the adjustment factor, results in near-zero weights that are 20× less than the weight assigned to the meta learner segmentation map. Therefore, we can conclude that the network disregards the adjustment factor in[7] when integrating the outputs of base and meta-learners. Based on this observation, we propose a method for reducing false positives caused by base classes that is both simpler and faster, resulting in a shorter training time with the same functionality and performance.

Table S2. Adjustment factor. The adjustment factor ψ used to combine information in the base learner of[7] has a mean $\mu_\psi < 0.1$ and a variance $\sigma_\psi < .05$. The small adjustment factor, in conjunction with the fact that these factors are multiplied by the weights which also have small values, leads to near-zero weight compared to the weight assigned to the meta learner segmentation map, which is 20× higher. Below we show the range of ψ after 10,000 episodes on Pascal-5^i and 70,000 episodes on COCO-20^i.

Dataset	Measure	f0	f1	f2	f3
Pascal-5^i	Average	0.087	0.083	0.082	0.081
	Min	0.051	0.039	0.047	0.044
	Max	0.181	0.199	0.162	0.169
COCO-20^i	Average	0.077	0.073	0.067	0.087
	Min	0.036	0.039	0.041	0.037
	Max	0.179	0.163	0.174	0.162

Learning intra-class similarity $S \to Q$: The impact of the Shannon entropy loss term

The 1st pass is supervised by,

$$\mathscr{L}_{combined} = \frac{1}{N} \sum_{n=1}^{N} [CE(BG_Q^1 \oplus FG_Q^1, Q_n^{gt}) - \kappa \mathscr{L}_{Sh}]$$

where $\kappa = 0.1$. The second term of $L_{combined}$ is the transductive loss term given by Shannon entropy $\mathscr{L}_{Sh}$ given by,

$$\mathscr{L}_{Sh} = \frac{1}{H \times W} \sum_{p=1}^{H \times W} (BG_Q^1(p) \oplus FG_Q^1(p)) log(BG_Q^1(p) \oplus FG_Q^1(p)) \quad (4)$$

where $p \in H \times W$ is pixel. The Shannon entropy encourages the network to have a polarised initial prediction with a high or low confidence area[8], which reduces the number of false positives.

Additional ablation

Table S3 shows the results of supervising the first pass with (bottom row) and without (top row) the Shannon entropy loss term $\mathscr{L}_{Sh}$. The experiments employ our method i.e. two-pass with B_{cls} + B_{sem}, for both 1-shot and 5-shot tasks on all folds of Pascal-5^i with Resnet-101 backbones for B_{cls} and B_{sem}. As it is evident, there is an improvement in the mIoU for each fold as well as the overall mIoU for both tasks.

Additional results

In Table S4, we present additional qualitative results, and visual comparisons with the state-of-the-art methods.

Table S3. Ablation. Supervising 1st *pass* with (bottom row - w) and without (top row - w/o) the Shannon entropy loss term $\mathscr{L}_{Sh}$.

		1-shot					5-shot				
Backbone	Method	f0	f1	f2	f3	mIoU	f0	f1	f2	f3	mIoU
Resnet-101	w/o $\mathscr{L}_{Sh}$	68.11	74.11	66.05	66.17	68.61	71.08	76.13	69.65	69.12	71.49
	w $\mathscr{L}_{Sh}$	69.22	74.49	67.20	66.81	69.43	73.28	77.01	69.94	69.64	72.46

Table S4. Qualitative results. The first and second columns show the support and query images, respectively, overlaid with the ground truth in red. The remaining columns show the predictions overlaid with a red.

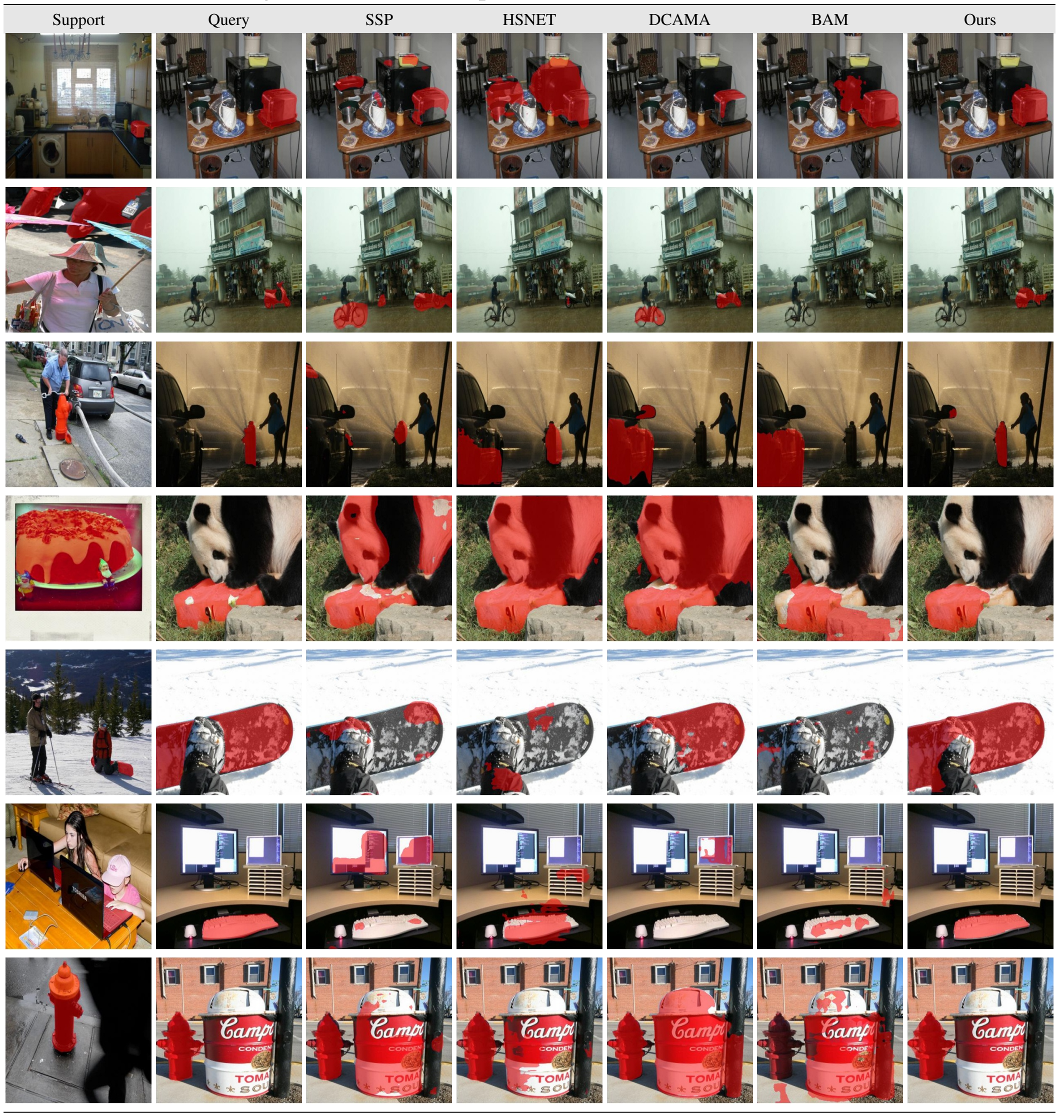

References

1. Wang, K., Liew, J. H., Zou, Y., Zhou, D. & Feng, J. Panet: Few-shot image semantic segmentation with prototype alignment. In *Proceedings of the IEEE Intl. Conf. on Computer Vision*, 9197–9206 (2019).
2. Zhang, C., Lin, G., Liu, F., Yao, R. & Shen, C. Canet: Class-agnostic segmentation networks with iterative refinement and attentive few-shot learning. In *Proceedings of the IEEE Conf. on Computer Vision and Pattern Recognition*, 5217–5226 (2019).
3. Fan, Q., Pei, W., Tai, Y.-W. & Tang, C.-K. Self-support few-shot semantic segmentation. In *European Conference on Computer Vision*, 701–719 (Springer, 2022).
4. Boudiaf, M., Kervadec, H., Imtiaz Masud, Z. & Piantanida, P. Few-shot segmentation without meta-learning: A good transductive inference is all you need? In *Proceedings of the IEEE Conference on Computer Vision and Pattern Recognition* (2021).
5. Teed, Z. & Deng, J. Raft: Recurrent all-pairs field transforms for optical flow. In *European conference on computer vision*, 402–419 (Springer, 2020).
6. Min, J., Kang, D. & Cho, M. Hypercorrelation squeeze for few-shot segmentation. In *Proceedings of the IEEE/CVF International Conference on Computer Vision*, 6941–6952 (2021).
7. Lang, C., Cheng, G., Tu, B. & Han, J. Learning what not to segment: A new perspective on few-shot segmentation. In *Proceedings of the IEEE/CVF Conference on Computer Vision and Pattern Recognition*, 8057–8067 (2022).
8. S. Dhillon, G., Chaudhari, P. & Ravichandran, A. A baseline for few-shot image classification. In *International Conference on Learning Representations(ICLR 2020)* (2020).
